# Synthesis and Morphological Control of VO_2_ Nanostructures via a One-Step Hydrothermal Method

**DOI:** 10.3390/nano11030752

**Published:** 2021-03-17

**Authors:** Ozlem Karahan, Ali Tufani, Serkan Unal, I. Burc Misirlioglu, Yusuf Z. Menceloglu, Kursat Sendur

**Affiliations:** 1 Faculty of Engineering and Natural Sciences, Sabanci University, Tuzla 34956, Istanbul, Turkey; alitufani@sabanciuniv.edu (A.T.); serkanunal@sabanciuniv.edu (S.U.); 2 Integrated Manufacturing Technologies Research and Application Center, Sabanci University, Teknopark İstanbul, Pendik 34906, Istanbul, Turkey; 3 Nanotechnology Research and Application Center, Sabanci University, Tuzla 34956, Istanbul, Turkey

**Keywords:** VO_2_ (M), phase transition temperature, hydrothermal synthesis, nanoparticle morphology

## Abstract

The morphology of nanostructures is a vital parameter to consider in components comprised of materials exhibiting specific functionalities. The number of process steps and the need for high temperatures can often be a limiting factor when targeting a specific morphology. Here, we demonstrate a repeatable synthesis of different morphologies of a highly crystalline monoclinic phase of vanadium dioxide (VO_2_(M)) using a one-step hydrothermal method. By adjusting the synthesis parameters, such as pH, temperature, and reducing agent concentration in the precursor, VO_2_ nanostructures with high uniformity and crystallinity are achieved. Some of these morphologies were obtained via the choice of the reducing agent that allowed us to skip the annealing step. Our results indicate that the morphologies of the nanostructures are very sensitive to the hydrazine hydrate (N_2_H_4_.H_2_O) concentration. Another reducing agent, dodecylamine, was used to achieve well-organized and high-quality VO_2_(M) nanotubes. Differential scanning calorimetry (DSC) experiments revealed that all samples display the monoclinic-to-tetragonal structural transition (MTST) regardless of the morphology, albeit at different temperatures that can be interpreted as the variations in overheating and undercooling limits. VO_2_(M) structures with a higher surface to volume ratio exhibit a higher overheating limit than those with low ratios.

## 1. Introduction

In pure form, oxides of vanadium (V_x_O_y_) have a rich variety of complex compounds and polymorphs. Because of the large range of oxidation states, V can attain (V^+2^, V^+3^, V^+4^, and V^+5^), it tends to form complex structures with anions [1,2] Among various crystal phases and hydrate structures of VO_x_ compounds, such as VO [3], V_2_O_3_ [4], V_3_O_7_.H_2_O [5], VO_2_.0.5H_2_O [6] and V_2_O_4_.0.25H_2_O [7], VO_2_ is a well-known and technologically important material with different polymorphs. Monoclinic VO_2_(M) has gathered great interest due to its ultrafast and fully reversible insulator-metal transition (MTST) between dielectric monoclinic VO_2_ (M) (space group P2_1_/c) and metallic rutile VO_2_(R) (space group P4_2_/mnm) phases at 68 °C [8]. It has also been a platform to study specific phenomena in phase transitions. For instance, the phase coexistence in a single crystalline solid-state media was reported experimentally in this system when in clamped form supported by theoretical predictions [9]. From a technological standpoint, VO_2_ possesses great potential for a wide range of application areas, such as electronic and optic switches [10,11], microbolometers [12], porous electrodes in Li-ion batteries [13], supercapacitor electrodes [14], programmable critical temperature sensors [15], memory devices [16], temperature sensors, and energy-efficient smart windows [17,18,19] Very recently, for example, Lee et al. [20] demonstrated that VO_2_, despite being in the metallic phase, has lower thermal conductivity, placing VO_2_ in a very unique and rare class of materials. Amid all the scientific activity focusing on applications, morphology has been demonstrated to be a dominant factor, particularly in optical applications where intrinsic material properties can couple to morphological scattering effects leading to the frequency dependence of spectral reflectance [21].

A significant portion of the existing literature on VO_2_ is based on the use of this material in the thin-film form [2,22,23,24]. In addition, VO_2_ nanostructures that are obtained using bottom-up approaches are also of interest due to their ease of integration with industrial processes and cost-effectiveness. The transition characteristics of VO_2_ nanostructures depend on their size and morphology [25,26], similar to many other systems that undergo structural symmetry-breaking transitions accompanied by an abrupt change in material properties [27,28]. The general tendency is that the smaller particle size reduces the MTST along with the decreasing enthalpy and entropy. Moreover, structures possessing cavities, such as hollow spheres and thin-walled nanotubes, also result in a lowering of the MTST [25,26]. Lowering of the MTST refers to the lowering of the temperature of the structural phase transition in VO_2_. A similar effect is well-known and reported in numerous studies focusing on the size and morphology dependence of phase transitions in systems, such as magnetic and ferroelectric materials. Depending on the nature of species present in the reaction medium and conditions, VO_2_ can be obtained in a wide range of morphologies, such as nanowires, nanofibers, nanorods, nanosheets (1D). Among these morphologies, nanotubes are of great interest because of their unique physical and chemical properties. A number of groups have reported the synthesis of VO_2_ nanotubes; however, the majority of these studies have utilized vanadium alkoxides as starting materials, which could be a quite costly procedure [29,30]. O’Dwyer et al. (2007) [29] used vanadium (V) triisopropoxide (VOTTP) as a precursor to a hydrothermal process at 180 °C, and VO_x_ nanotubes are grown on a nanourchin structure. The nanotubes are ~2 µm in length with inner diameters of 20–30 nm, and individual nanourchin is ~10–12 µm in length. O’Dwyer et al. [30] again obtained urchin-like nanotubes using VOTTP at 180 °C lasting for 7 days by changing some reaction procedures. Such nanotubes are typically 100–120 nm in diameter with inner open diameters of 70–80 nm. However, other groups have reported the use of vanadium oxides instead of alkoxides [31,32] to demonstrate cost-effective synthesis procedures for this material. Mai et al. [33] presented the synthesis of 1D vanadium oxide nanowires by electrospinning using inorganic NH_4_VO_3_ as a precursor and stated that the growth of NH_4_VO_3_ nanorods on the surface of NH_4_VO_3_/PVA composite nanowires before annealing is crucial for the production of hierarchical vanadium oxide nanowires. However, low-dimensional nanomaterials might have the disadvantage of dispersibility (self-aggregation) due to their higher surface energy. Highly ordered three-dimensional (3D) vanadium oxide nanomaterials in the form of urchins [29,34,35], flowers [36,37] and tubes [29,31,32] have been synthesized to overcome this problem. Pan et al. [34] presented the different morphologies of 3D vanadium oxide (VO_x_) microstructures (urchin-like micro flowers, nanosheet-assembled microflowers, nanohorn-structure microspheres) that were synthesized using a VOC_2_O_4_ precursor using the solvothermal synthesis method. They stated that the concentration of the precursor solution has an important influence on the morphologies of the products. Fei et al. [36] report the 3D flower-like sodium ammonium vanadium bronze ((NH_4_)_0.26_Na_0.14_V_2_O_5_) nanostructure by hydrothermal synthesis method at 180 °C for 24 h. The thickness of platelets is less than 50 nm as estimated from the magnified SEM, and one urchin size is about ~9–10 µm. Yu et al. [37] noted that the Cu-doped V_2_O_5_ microflowers were synthesis with a hydrothermal approach followed by heat-treatment in air. They represent the formation of highly uniform discrete flower-shaped structures with an average diameter of ~10 µm.

All of the aforementioned studies obtained different morphologies of VO_x_ employing different synthesis techniques. In the realm of discovering their potential, simple and reproducible yet scalable and versatile synthesis strategies are needed to obtain highly crystalline VO_2_ with controllable morphologies that eliminate the need for a post-annealing step. Previous strategies generally have relied on the choice of the method, such as sol–gel synthesis [38], controlled oxidation and sputtering [39], chemical vapor deposition [40], and physical vapor deposition [41] that ends up with various morphologies of VO_2_ structures. Hydrothermal synthesis is another effective method providing a rich parameter space as a toolbox to obtain high-quality structures in substantial amounts [42,43]. Compared to other synthesis methods, hydrothermal synthesis is a cost-effective and readily scalable procedure that provides the desired phases for various metal oxide nanostructures. In general, metastable VO_2_(B) (monoclinic) [44,45] and VO_2_(M) phases obtained by this method require a post-annealing/heating process to convert the VO_2_(B) phase to VO_2_(M); however, direct synthesis of the monoclinic phase of VO_2_ by the hydrothermal synthesis has seldom been reported [44]. Crucial parameters, such as precursor concentration, reaction temperature, time, and pH, have been previously studied to investigate their effect on the synthesis of high purity VO_2_ in the M phase employing cost-effective and repeatable techniques [44].

Here, we demonstrate a one-step hydrothermal synthesis procedure where all mentioned morphologies can be achieved through adjusting the synthesis parameters and with uniformity and crystallinity. The proposed single-step hydrothermal method can eliminate the annealing step in the synthesis of VO_2_ powders, wherein we can control the morphology via the process parameters. This is a great improvement over many earlier works where multistep processes have been used that often require a high-temperature annealing step, which some applications cannot tolerate due to the detrimental effect of high temperatures during fabrication. Some of these morphologies can be obtained via the choice of the reducing/directing agent, eliminating the need for an annealing step. The effect of morphology on the MTST temperature of synthesized VO_2_ nanostructures was also compared using DSC. The calorimetric measurement is the best-suited method to characterize a bulk property such as a phase transition and is a method widely used to identify many types of phase transitions. In addition, we identify the overheating and undercooling effects quite effectively arising from the energy barriers (likely related to the elastic strains that develop at the interfaces between the monoclinic and tetragonal phases) in the transition.

## 2. Materials and Methods

### 2.1. General Experimental

Vanadium pentoxide (V_2_O_5_, 99.99%-Sigma, Schelldorf, Germany), hydrazine hydrate (24–26% solution in water), ethanol (absolute, 99%), hydrochloric acid (36.5–38%), dodecylamine (98+%) and potassium hydroxide were purchased from Sigma-Aldrich (Schelldorf, Germany). All the chemicals were used as received without further purification. The synthesis of different morphologies of VO_2_(M) was carried out utilizing a modified version of a hydrothermal method earlier performed by Gui et al. [46]. We preferred this procedure because it bypasses the need for specific equipment connected with gas-phase systems and high temperatures required to enable the chemical reactions. Furthermore, this procedure has the potential to allow the fabrication of high-quality nanomaterials in large amounts with precise control over morphology without the requirement of further annealing. We provide in the sections below details as to the specific morphologies obtained via varying the synthesis conditions.

Hydrazine was essential for the formation of VO_2_(M). When only KOH solution was applied for the precipitate form without hydrazine, VO_2_(B) nanomaterial formed without any VO_2_(M) [47]. It was hypothesized that hydrazine performed a significant role as a coordinating ligand and reducing agent, helping the formation of VO_2_(M). Hydrazine has been reported as a structure-directing agent for VO_2_ hydrate [48]. When small quantities of surfactants, for instance, CTAB, PVP or organic ligands, such as oxalic acid was incorporated as additives, VO_2_(B) selectively formed rather than VO_2_(M). This observation points out the likelihood that the presence of small quantities of organic molecules in the reaction solution promotes the formation of VO_2_(B) [47].

In recent studies, excess oxalic acid was also employed as another reducing agent on the hydrothermal synthesis of VO_2_(M) utilizing V_2_O_5_. However, when oxalic acid is used for the reduction of V_2_O_5_ to VO_2_ by hydrothermal synthesis, CO and CO_2_ gas is released as a side-product. On the other hand, N_2_ gas is released when hydrazine hydrate is used as a reducing agent. Being a strong reducing agent, when hydrazine is added into the V_2_O_5_ solution, a color change from dark yellow to black occurs, indicating the formation of V compounds with lower valence states. The chemical reaction for the reduction of V (V) to V (IV) is given below:

(1) $$V_{2}O_{5}+\;\frac{1}{2}{\;N}_{2}H_{4}=2{\mathrm{VO}}_{2}\;+\;\frac{1}{2}{\;N}_{2\;}↑+{\;H}_{2}O$$

The molar ratio of V_2_O_5_ to hydrazine and the reaction temperature are critical factors for the synthesis of highly crystalline VO_2_ nanomaterials [49]. If the reaction temperature is not high enough, a post-heating process may be needed to produce crystalline materials. A similar heat-treatment would be required if the amount of hydrazine used in the hydrothermal synthesis is insufficient, and the variation of hydrazine concentration in a narrow range has a critical impact on the chemical valance of V.

Here, we demonstrate the effect of the concentration of hydrazine hydrate on the morphology and the transition temperature of VO_2_(M). After the synthesis, all samples were characterized by X-ray diffraction (XRD, θ−2θ values of 20–70° at 0.02 step size with Cu-K_α_ radiation on a Bruker AXS-D8 diffractometer) and Raman spectroscopy (Renishaw Reflex Raman microscope with 532 nm laser), for phase identification followed by field-emission scanning electron microscopy (FE-SEM, Leo-G34 SEM at 4 kV) to determine the morphology. Differential scanning calorimetry (DSC, TA Q−2000 equipment using N_2_ atmosphere) was carried out to identify the MTST of the samples.

### 2.2. Synthesis of Asterisk-Like, Urchin-Like and Spherical Shaped VO_2_ (M)

To achieve various morphologies of VO_2_(M), 0.005 mol (0.90 g) V_2_O_5_ was added to 80 mL water and subjected to ultrasonication with a probe for 30 min (Q700, QSONICA, 40% amplitude, pulse on 5 s, pulse off 5 s) until a dark yellow solution was obtained. Then 0.015 mol (0.84 g) KOH was added to this solution dropwise at room temperature, and the solution color turned from blurry to clear yellow solution after stirring for 10 min. 0.0437 M (0.17 mL) hydrazine hydrate was added dropwise into the clear yellow solution in an ice bath, and the color turned from yellow to green then to brown during the hydrazine hydrate addition. The pH of the solution was set to 3.3–3.5 with hydrochloric acid, and the color turned black. After the reaction mixture was stirred for 2 h at room temperature, it was transferred into a Teflon-lined, 250 mL autoclave reactor and placed at 200 °C for 24 h. Following this step, the autoclave reactor was cooled to room temperature, and the final reaction mixture containing the product was centrifuged 3 times with water to obtain black precipitates asterisk-like VO_2_ nanostructures that were dried in an oven at 80 °C for 12 h. Thereafter, noting that the samples having an asterisk-like morphology did not fully convert to VO_2_(M), they were additionally annealed at 500 °C for 2 h under highly pure N_2_ with the heating rate of 10 °C min^−1^.

The amount of hydrazine hydrate in the above synthesis procedure was increased two or three times (0.087 and 0.131 M) to see the effect of concentration of reducing agent on the morphology and urchin-like and multifaceted spherical VO_2_(M) nanoparticles that were obtained, respectively, without annealing. On the other hand, when the hydrazine hydrate amount was increased four or five times, the morphology of VO_2_ remained in a multifaceted spherical nanostructure form. Table 1 represents the summary of monoclinic VO_2_ morphologies and the corresponding experimental conditions.

### 2.3. Synthesis of Nanotube Shaped VO_2_ (M)

A total of 0.55 g V_2_O_5_ was added to 40 mL water and subjected to ultrasonication with a probe for 30 min. Another solution of 0.9 g dodecylamine in 40 mL ethanol was prepared. Both solutions were mixed together, which reached a pH of 6.0–6.5 and was stirred for 5 h at room temperature. The mixture was then transferred into a Teflon-lined 250 mL autoclave reactor and kept at 150 °C for 5 days. Afterward, the autoclave reactor was cooled down to room temperature, and the reaction mixture was centrifuged 3 times each with water and ethanol. Upon centrifugation, the obtained VO_2_ powder was dried in the oven at 200 °C for 5 h.

In the literature, N_2_H_4_ is known as an effective reduction agent [50], and various mechanism, including bubble formation leading to various morphologies, has been attributed to different amounts of N_2_H_4_ [25]. We designed experiments centered around identifying the effect of N_2_H_4_ concentration on the morphologies of powders that were in-tended functional components of a composite structure. The mechanisms involving N_2_H_4_ were further discussed in the literature by demonstrating the effects on the synthesis of VO_2_ nanoparticles in a hydrothermal assisted homogeneous precipitation approach. While N_2_H_4_is known to be an effective reducing agent, morphologies have been obtained in our work via the molecules that were intuitively considered to cause a highly oriented formation of the VO_2_ structure.

On the other hand, the primary role of dodecylamine on the nanotube formation, VO_2_ replicates the molecular anisotropy of the dodecylamine resulting in a highly oriented growth. In addition, it facilitates the reduction of V(V) to V(IV). Thereby, a conformal growth along alkyl amine chains is realized, as reported in the manuscript. In essence, dodecylamine acts as a template for VO_2_ to form along its axis [32].

## 3. Results and Discussions

**Figure 1:**
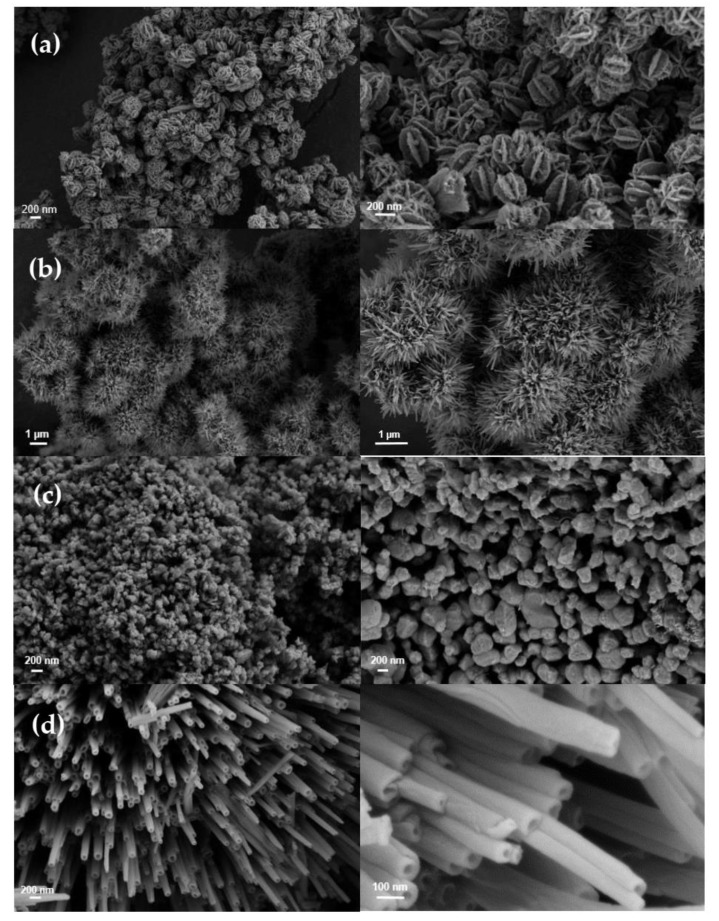
SEM images of various VO_2_ nanoparticles: (**a**) asterisk-shaped (**b**) urchin-like, (**c**) multifaceted spherical (**d**) nanotube. Images on the right column represent the magnified versions of those on the left column.

To demonstrate the impact of the synthesis conditions on the morphology of the VO_2_ powders, we first provide the FE-SEM images of the samples synthesized with varying hydrazine hydrate concentrations (0.0437, 0.087 and 0.131 M) in Figure 1. Asterisk-shaped VO_2_ crystal structures starting to grow branches from hexagon-shaped crystals were identified in Figure 1a. Such formations have diameters ranging from 150 nm to 200 nm. In Figure 1b, urchin-like VO_2_ structures are observed that were obtained using 0.087 M hydrazine hydrate. Numerous μm-scale urchin-like VO_2_ structures are visible, with radial tubular arrays having lengths of 250–300 nm. The formation of this latter structure was due to the effect of the reducing agent whereby a chelated structure with V_2_O_5_ was expected to form to produce an urchin-like structure [34]. In Figure 1c, multifaceted nano-spherical VO_2_ crystal structures with diameters ranging from 100 nm to 200 nm are visible using 0.131 M hydrazine hydrate. FE-SEM images of nanotubular VO_2_ structures are shown in Figure 1d. The inner diameters of tubes were around 30–35 nm, and outer diameters were around 80–85 nm.

In Figure 1d, all nanotubes were aligned parallel to form a structure similar to the urchin-like one but much more extended in a single direction. The formation of this structure is due to the effect of dodecylamine, whereby a chelated structure with V_2_O_5_ forms leading to a nanotube structure. The lamellar structure of VO_2_(M) promotes a mechanism whereupon the amine-intercalated slabs roll up under hydrothermal treatment [51]. In this process, the amine surfactant molecules accumulate and condense between the vanadate layers. Upon heating, this results in more-ordered structures, with the lamellar sheets finally rolling into nanotube structures. The final black material is a result of the reduction of V^5+^ to V^4+^ by amine decomposition [52].

**Figure 2:**
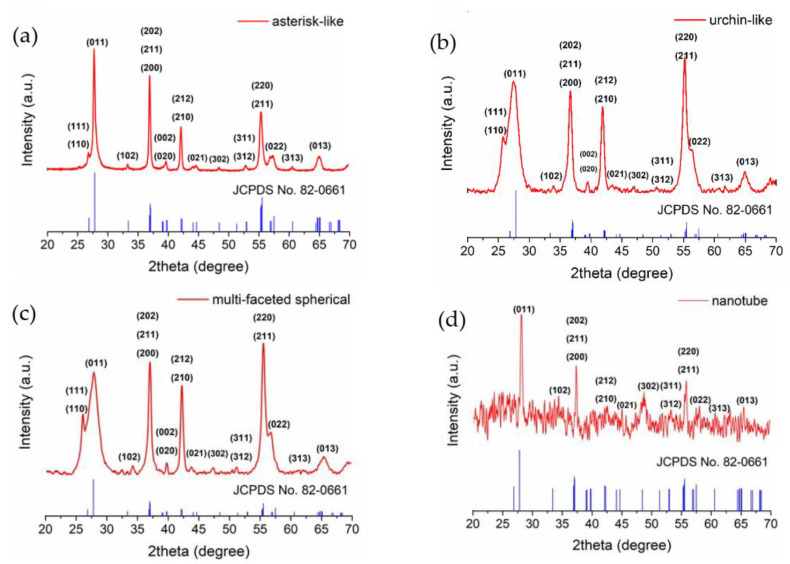
XRD spectrum of VO_2_ nanoparticles with different morphologies: (**a**) asterisk-like (**b**) urchin-like, (**c**) multifaceted spherical (**d**) nanotube. Standard XRD pattern is plotted in blue.

The XRD spectra of the samples are displayed in Figure 2. Note that for samples synthesized with 0.087 and 0.131 M hydrazine (urchin-like, multifaceted spherical, respectively), the annealing procedure was not necessary. In all synthesis conditions, the monoclinic VO_2_ phase was observed. For the sake of completeness, the XRD data of the black powder sample of hydrated VO_2_ (VO_2_.H_2_O) itself is provided in Figure S1 (using 0.0437 M hydrazine hydrate (asterisk-like)), which later converts entirely to VO_2_(M). Following the hydrothermal synthesis, the powders with the asterisk-like morphology were subjected to annealing at 500 °C under nitrogen for 2 h to obtain a pure VO_2_(M) phase (Figure 2a). Some works report that after the hydrothermal synthesis, the hydrated VO_2_ (VO_2_.H_2_O) has been dehydrated at the 230 °C, which was accompanied by a broad endothermic peak that was attributed to the loss of water molecules followed by a transition from β-phase to rutile/monoclinic phase at 320 °C. This transition upon heating is identified by an exothermic peak [46]. Tsang et al. [53] has reported that the β-phase of VO_2_ is observed at 300 °C and rutile phase started to form at 330 °C and complete phase transition occurs at 500 °C, whereas Gui et al. [54] have reported that the β-phase of VO_2_ might be stable all the way until 620 °C. In our study, we performed annealing, when needed, at 500 °C in a nitrogen atmosphere for 2 h. Looking at Figure 2a for the asterisk-like structure, the characteristic peak of the M-phase at 2θ = 27.711° is present, and V_2_O_5_ that is stabilized at higher temperatures is completely transformed into VO_2_(M). The XRD pattern of the urchin-like VO_2_ structures is provided in Figure 2b, which was obtained using 0.847 M hydrazine concentration. A characteristic peak of the monoclinic VO_2_ phase is clearly visible at 2θ = 27.425° and again, no indication of the presence of the β-phase is evident. In fact, the increase in the hydrazine concentration led to multifaceted spherical nano VO_2_ particles, for which the XRD spectrum is given in Figure 2c, verifying the formation of the pure monoclinic VO_2_ (M) phase for this morphology. Replacing the hydrazine with a dodecylamine reducing agent, which is a long-chain alkylamine, imposed the growth of tube-like VO_2_ structures without the requirement of a post-annealing, resulting in VO_2_(M) nanotubes whose XRD data are displayed in Figure 2d. Here, the significant loss of the intensity of all peaks (visible from the increased signal-to-background ratio) is likely due to the strong anisotropy of the crystal orientation, eventually resulting in a reduction of the volume that scatters the incident radiation, i.e., a result of a loss of scattering cross-section. Despite the noisy background due to signal intensity loss, the main sharp peaks correspond to VO_2_(M), which matches well with that of standard JCPDS card No. 82–0661 [48,55]. We would like to draw attention to the fact that FWHM of the XRD peaks varies as a function of a sample morphology, implying the likelihood of the high aspect ratio structures containing fewer structural variations, such as domains and defects.

**Figure 3:**
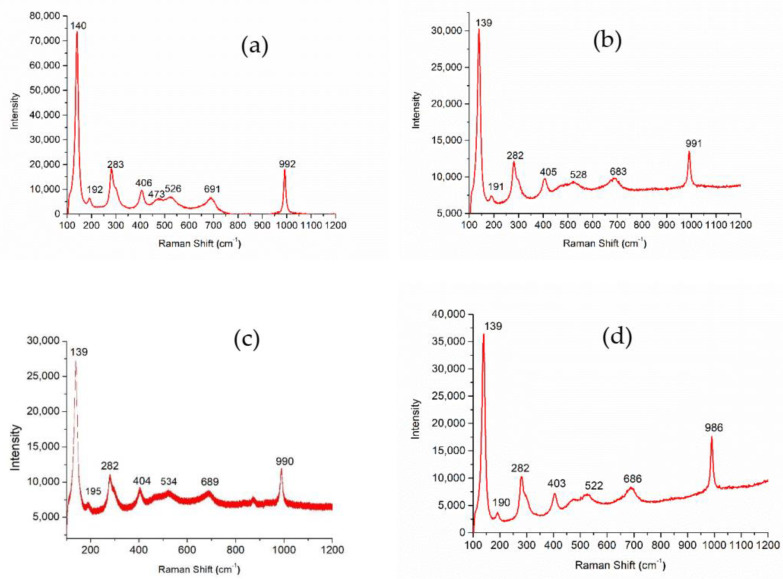
Raman spectra of VO_2_ nanoparticles with different morphologies (**a**) asterisk-shaped (**b**) urchin-like, (**c**) multifaceted spherical (**d**) nanotube.

To probe any further possible differences in the synthesized VO_2_ structures, a Raman spectroscopic analysis of each VO_2_ sample with the aforementioned morphologies was carried out, as shown in Figure 3. Specifically, Raman spectroscopy was utilized to verify the M phase of VO_2,_ particularly in the nanotubular structure, as the XRD signal from this sample was relatively low, as shown in Figure 2d. All Raman active modes of VO_2_(M) were observed in all samples (Figure 3a–d). A strong band at 140 cm^−1^ corresponds to V-O-V bending modes and external modes (bending/wagging), while 992 cm^−1^ is associated with V=O stretching of distorted octahedral and distorted square-pyramids. The bands at 406 cm^−1^ and 473 cm^−1^ are attributed to bending vibrations of the bridging V-O-O bond. The bands at 192 cm^−1^ and 283 cm^−1^ are associated with bending vibrations of V=O bonds. The band at 526 cm^−1^ originates from the V_3_-O stretching mode of edge-shared oxygen in common with three pyramids, while the band at 691 cm^−1^ is dedicated to V_2_-O stretching mode, which is due to corner-shared oxygen common to two pyramids [56].

**Figure 4:**
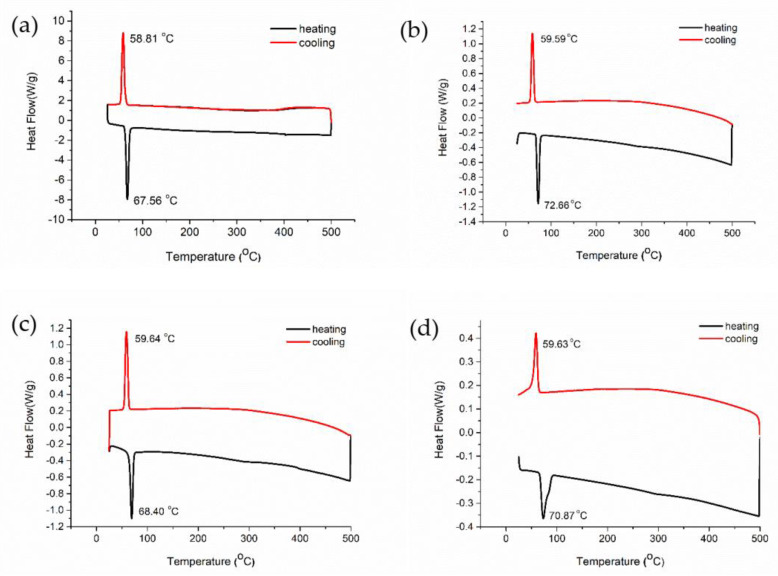
DSC graphs of (**a**) asterisk-like VO_2_ (**b**) urchin-like VO_2_ (**c**) multifaceted spherical-like VO_2_ (**d**) nanotubular VO_2._

In order to investigate if different morphologies impact the MTST temperature of VO_2_ (M), differential scanning calorimetry (DSC) analyses were carried out on all samples. The DSC data are provided in Figure 4. During the DSC analysis, the temperature was increased from 25 °C to 500 °C and then cooled down from 500 °C to 25 °C at a rate of 10 °C/min. After the heating of VO_2_.H_2_O at 520 °C for 2 h under N_2_, pure monoclinic VO_2_ (asterisk-like) was obtained (See Figure 2a), and its DSC analysis is shown in Figure 4a. During the cooling of asterisk-like VO_2,_ an endothermic peak is visible at 58.81 °C, followed by an exothermic peak at 67.56 °C during heating. The absence of any other peaks confirms that a pure monoclinic phase of VO_2_ with a clear MTST temperature was obtained.

DSC graph of urchin-like VO_2_ is given in Figure 4b, which shows an endothermic peak at 72.66 °C during heating and an exothermic peak at 59.59 °C during cooling. Multifaceted spherical-like VO_2_ sample shows an endothermic peak at 68.40 °C upon heating and an exothermic peak at 59.64 °C upon cooling without any other phase transitions, as shown in Figure 4c. Figure 4 points out the striking difference in the heating and cooling transition temperatures, almost 9 °C, revealing a strong hysteresis that is a characteristic of first-order phase transitions. Such hysteresis, by definition, occurs due to the barriers to nucleation of the second phase in a matrix of the parent phase. Overcoming the barrier, which is basically the energy of the interface between the rutile and monoclinic phases, requires overheating and undercooling for the transition to occur. The thought that MTST is expected to be a function of size should not be confused with the variations in undercooling and overheating limits of structural symmetry-changing phase transitions. In a DSC experiment, it is questionable whether the system reaches an almost equilibrium state at every temperature. Thus, undercooling (during cooling) and overheating (during heating) is required to initiate the nucleation of the new phase, which is prone to interpretation as “the change in the transition temperature” of the system. A change in the transition temperature of any system should be accompanied by a change in the undercooling and overheating limits, which are within the same range for all samples with different morphologies in this study. Keeping in mind that DSC analysis reveals the undercooling and overheating behavior, we, therefore, interpret the variations in the temperature peaks in our DSC runs as originating from the number of nucleation and growth sites available to the new phase (rutile, while heating and monoclinic phase, while cooling) that is expected to depend on the geometry, i.e., morphology. From the experimental data, it is noted that the overheating limit, in particular, has a tendency to increase with large aspect ratio features in the VO_2_ structure, signaling a larger barrier to nucleation and growth of the new rutile phase in VO_2_(M). The interplay between atomic bonding and the phonon dynamics [25,26] have been cited to be the cause of the size dependence of the MTST, but it is also important to bear in mind that small variations in transition temperatures need to be discussed within nucleation and growth process kinetics. We argue so given that experimental measurements on such systems are conducted in finite amounts of time, whereas claiming changes in thermodynamic quantities need to assure that kinetic effects due to energy barriers, such as those for nucleation and growth of a new phase in a matrix, are excluded. The MTST behavior near T*_c_* for the asterisk-like and multifaceted spherical VO_2_ nanoparticles are quite identical, which is attributed to the identical diameters and morphologies of both structures.

Figure 4d displays the DSC graph of nanotubular VO_2_ that is geometrically similar to the urchin-like nanostructure, where both of them have a higher aspect ratio than spherical and asterisk-like VO_2_. According to Figure 4, the increase in the MTST temperature of the urchin-like structure compared to asterisk-like and multifaceted morphologies during heating could either be interpreted as a thermodynamic change in the transition or a change of the overheating/undercooling limits. The former can be expected due to more difficult nucleation of the rutile phase inside the monoclinic one due to geometrical restrictions. An effective increase in the energy barrier for nucleation will require further overheating to transform the structure from the monoclinic to the metallic rutile. A high aspect-ratio geometrical state of the parent phase can be thought to be favoring or imposing a high anisotropy constraint on the phase to be nucleated that restricts the “atomic plane” options of the interface to form, resulting in the nucleation of the new phase with a higher barrier. It is also obvious that a single phase transition during heating and cooling presents a single α-phase of VO_2_(M) that is obtained during synthesis. In a study by Wang and coworkers, it has been noted that hollow cavities of nanomaterial structures have a significant effect on the crystal transition temperature of VO_2_(M) apart from the size effect [25]. The crystal transition temperature of nanotubular VO_2_ was found to be 70.87 °C, lower than urchin-like VO_2_ transition at 72.66 °C, which can be a result of hollow cavities of nanostructures (Figure 4d).

Overall, DSC results displayed in Figure 4 confirm that the overheating limit is relatively more sensitive to morphological changes in the VO_2_ nanostructures than the undercooling limit, where we have not observed any significant variations. Contrary to the intuition, structures with a higher surface to volume ratio exhibit a higher overheating limit than those with low aspect ratios. We find this counter-intuitive because surfaces are often designated as stress-free boundary conditions in elasticity and, therefore, upon heating, a newly formed nucleus of the low symmetry M phase inside a rutile matrix deforms less volume in a high aspect ratio structure than if it were inside a spherical structure. As one would expect lower elastic energy in the former (high aspect ratio) structure accumulates in the volume upon the appearance of the new phase, it corresponds to a lower barrier to nucleation; hence a lower overheating is required. This is the very argument that we base the morphological dependence of our nanostructures; however, the DSC experiments reveal an opposite trend: The spherical-like structures exhibit lower overheating limits with respect to the urchin-like and nanotubular structures, demonstrating the sensitivity of the overheating limit to the morphology. It is well-known that in high surface-to-volume ratio structures, such as fibers, the defect content is lower due to restrictive volume and cost of the energy of the “defect field”. Defects are well-known to act as nucleation centers in phase transitions. Therefore, a reduced number of defect site concentrations is expected to lead to a lower nucleation rate of the R phase accompanied by an increased energy barrier. The fact that the undercooling limit is almost independent of morphology is likely due to the large barriers imposed by the elastic misfit between the low symmetry M phases that nucleate inside a higher symmetry rutile phase, making this process insensitive to morphology.

## 4. Conclusions

In this paper, we report the synthesis and characterization of various morphologies of VO_2_(M) nanocrystals using a one-step hydrothermal treatment of hydrolyzed precipitate from V_2_O_5,_ in which N_2_H_4_.H_2_O is employed as a reducing agent. Using this approach, the synthesis of asterisk-like, urchin-like and multifaceted spherical nanostructures of highly crystalline and uniform monoclinic vanadium dioxide was demonstrated. In order to achieve well-organized nanotubes, we turned our attention to dodecyl amine both as a reducing and a structure-directing agent, owing to its long molecular chains. As a result, high-quality, well-organized and directed VO_2_ nanotubes with a uniform size distribution were synthesized. We identified the transition temperatures of all the structures where an apparent dependence of the transition temperatures on morphology existed. Such changes are attributed to the nucleation and growth kinetics that shifts the system towards an overheating or undercooling at the transition depending on the surface area of a given morphology. Therefore, we emphasize that the changes in the peak positions of the DSC data with varying size or morphology for a solid–solid transition of the type reported here do not necessarily imply a change in the intrinsic thermodynamic transition temperature but rather a change in the nucleating barriers to the forming phase. Our DSC results indicate that the overheating limit is relatively more sensitive to morphological changes in the VO_2_ nanostructures than the undercooling limit, where we did not observe any significant variations. We believe that the method prescribed herein has great potential, especially for optical applications of VO_2,_ where the combined effects of morphology-driven scattering and size-induced transition effects can be tailored.

## Figures and Tables

**Table 1:** Summary of monoclinic VO_2_ morphologies and corresponding experimental synthesis conditions.

Morphology	Reducing Agent	Amount of Reducing Agent	Acid	Temperature (°C)	Time (h)
Asterisk-like *	N_2_H_4_.H_2_O	0.17 mL	HCI	200	24
Urchin-like	N_2_H_4_.H_2_O	0.34 mL	HCI	200	24
Multi faces spherical	N_2_H_4_.H_2_O	0.51 0.68 and 0.84 mL	HCI	200	24
Nanotube	Dodecylamine	0.9 g	-	150	120

* It was annealed at 500 °C for 2 h under highly pure N_2_ with the heating rate of 10 °C min^−1^.

## Data Availability

The data that support the findings of this study are available from the corresponding author upon reasonable request.

## Supplementary Materials

The following are available online at https://www.mdpi.com/2079-4991/11/3/752/s1, Figure S1: XRD pattern of VO_2_.H_2_O precursor before annealing (JCPDS #13−0346) [48].
