# Supplementary material for: Synthesis and Morphological Control of VO2 Nanostructures via a One-Step Hydrothermal Method
Source: Nanomaterials (Basel). 2021 Mar 17;11(3):752. doi: 10.3390/nano11030752 (PMC8002504; doi:10.3390/nano11030752)
Supplement: Supplementary file 1 [file nanomaterials-11-00752-s001.pdf]

Supporting Information:

Synthesis and Morphological Control of VO_2 Nanostructures via a One-Step Hydrothermal Method

Ozlem Karahan [1,*], Ali Tufani [1], Serkan Unal [1,2], I. Burc Misirlioglu [1,2,*], Yusuf Z. Menceloglu [1,2,3,*] and Kursat Sendur [1,*]

1 Faculty of Engineering and Natural Sciences, Sabanci University, Tuzla 34956, Istanbul, Turkey; alitufani@sabanciuniv.edu (A.T.); serkanunal@sabanciuniv.edu (S.U.)
2 Integrated Manufacturing Technologies Research and Application Center, Sabanci University, Teknopark İstanbul, Pendik 34906, Istanbul, Turkey
3 Nanotechnology Research and Application Center, Sabanci University, Tuzla 34956, Istanbul, Turkey
* Correspondence: ozlem.karahan@boun.edu.tr (O.K.); burc@sabanciuniv.edu (I.B.M.); yusufm@sabanciuniv.edu (Y.Z.M.); sendur@sabanciuniv.edu (K.S.)

Supplementary Materials: The following are available online at https://www.mdpi.com/2079-4991/11/3/752/s1, Figure S1: XRD pattern of $VO_2.H_2O$ precursor before annealing (JCPDS #13-0346) [48].

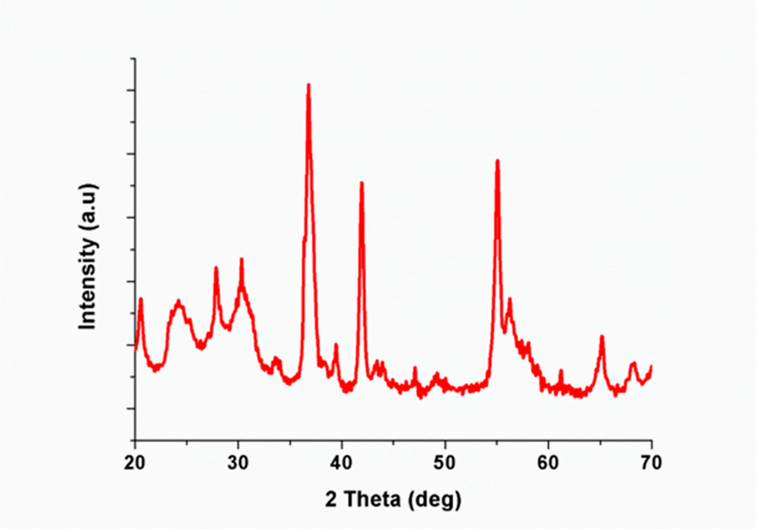

Figure S1. XRD pattern of $VO_2.H_2O$ precursor before annealing (JCPDS #13-0346) [48].
